# Supplementary material for: Uptake of Tropheryma whipplei by Intestinal Epithelia
Source: Int J Mol Sci. 2023 Mar 24;24(7):6197. doi: 10.3390/ijms24076197 (PMC10094206; doi:10.3390/ijms24076197)
Supplement: Supplementary file 1 [file ijms-24-06197-s001.zip › S3.pdf]

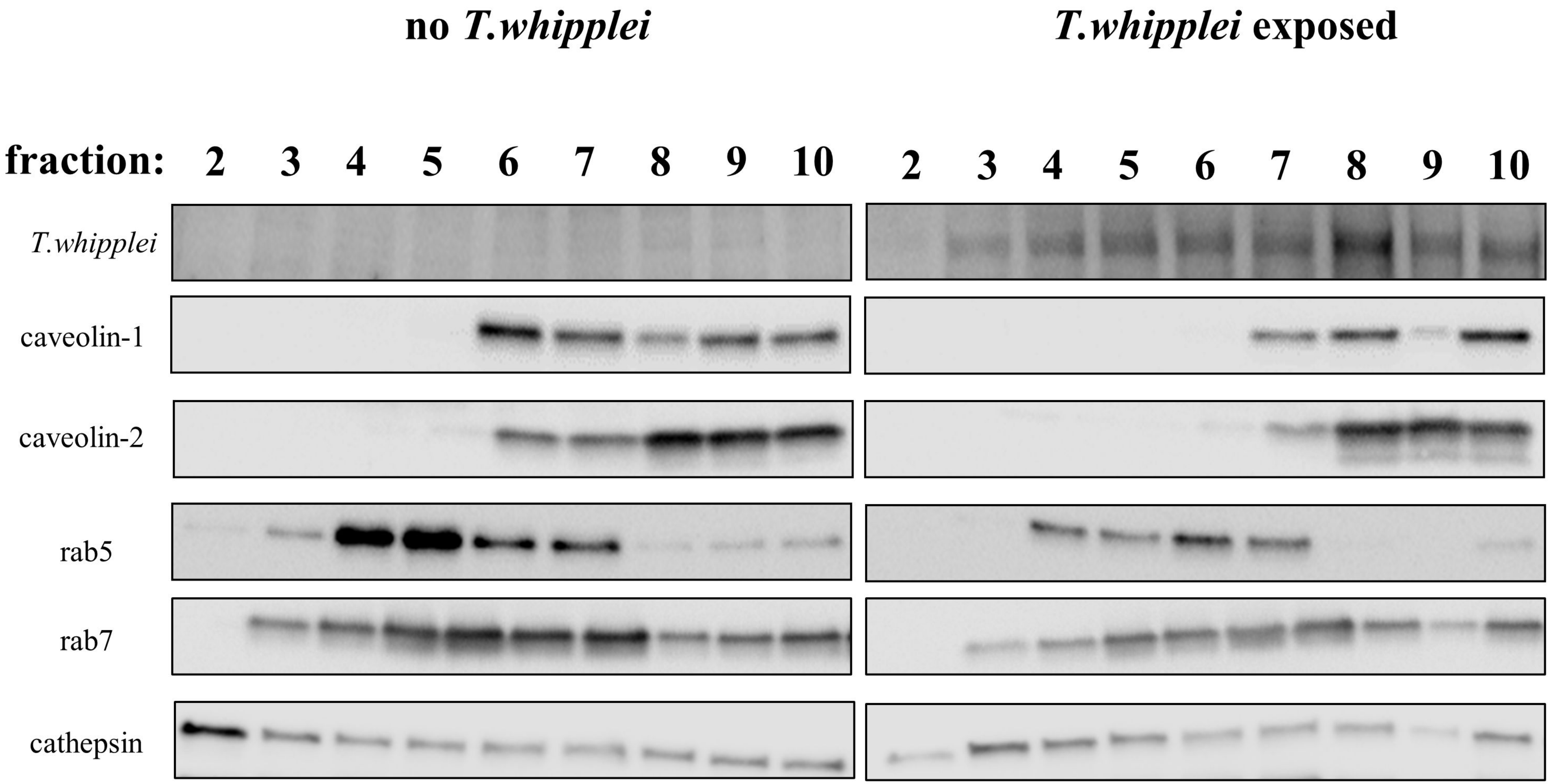

no *T.whipplei*
T.whipplei exposed
fraction: 2 3 4 5 6 7 8 9 10
2 3 4 5 6 7 8 9 10
T.whipplei
caveolin-1
caveolin-2
rab5
rab7
cathepsin
