# Supplementary material for: Uptake of Tropheryma whipplei by Intestinal Epithelia
Source: Int J Mol Sci. 2023 Mar 24;24(7):6197. doi: 10.3390/ijms24076197 (PMC10094206; doi:10.3390/ijms24076197)
Supplement: Supplementary file 1 [file ijms-24-06197-s001.zip › Supplemental Material Legend.pdf]

Supplemental Material Legends

Suppl. figure S1: Example of a flow cytometric analysis of uptake of *T. whipplei* by Caco-2 cells. All specimens were incubated with vital *TW*, fixed in 4% paraformaldehyde followed by staining with rabbit anti-*TW* followed by donkey-anti-rabbit-Alexa647. **A:** control with no further additives. **B, C, D:** sample with inhibitors of actin polymerization Latruculin A (B), Cytochalasin D (C), and Genistein (D).

Suppl. figure S2: Example of a flow cytometric analysis of uptake of *T. whipplei* by Caco-2 cells following transfection with either Eps mutant, wildtype Eps, K44A-Dynamin or wildtype Dynamin. WT, wildtype. All specimens were incubated after transfection with vital *TW*, fixed in 4% paraformaldehyde followed by staining with anit-PARP-PE (PARP, Poly (ADP-ribose) polymerase), rabbit anti-*TW* followed by donkey-anti-rabbit-Alexa647. Caco-2 cells were gated in a SSC/FSC blot, followed by gating on GFP^+ transfected cells and analysis of *TW* and PARP expression.

Suppl. figure S3: Subcellular fractionation. Western blotting of *TW*-exposed vs. non-exposed Caco-2 cells after subcellular fractionation by ultracentrifugation of lysates on sucrose density gradients. *TW*-positive fractions are identified by parallel immunoblotting of various marker proteins, including caveolin-1 and caveolin-2, rab5 (for early endosomes), rab7 (late endosomes), and cathepsin (lysosomal compartment). The compartments containing *TW* were recovered in fractions 3-10 of the sucrose gradient, with a focus on fraction 8 (which has a low expression of rab5).

Suppl. figure S4: Domain structure of caveolin-1 and caveolin fragments. Cav1, caveolin-1; AA, amino acids.

Suppl. figure S5: Example of a flow cytometric analysis of uptake of *T. whipplei* by Caco-2 cells. All specimens were incubated with vital *TW*, fixed in 4% paraformaldehyde followed by staining with rabbit anti-*TW* followed by donkey anti-rabbit-Alexa647. **A:** control with no further additives. **B, C:** sample with inhibitors of small GTPases EHT 1864 (B) and FTS (C).

Suppl. figure S6: Western blot of total lysates for caveolin-1 and caveolin-2 after exposure of Caco-2 cells to *TW*. Representative western blot.

Suppl. figure S7: Impact of heat inactivation of *T. whipplei* on endocytosis. Caco-2 cells were exposed to either *TW* or heat-inactivated *TW* (100°C, 90 min). Epithelial uptake was assessed by flow cytometry. Heat inactivation of *TW* reduced epithelial uptake. Single values, median and minimum to maximum, n=5-6. ***$p<0.001$.

Suppl. figure S8: Example of a flow cytometric analysis of uptake of *T. whipplei* by Caco-2 cells. **A:** isotype control stained with rabbit serum and donkey anti-rabbit-Alexa647. **B, C:** Caco-2 incubated with vital *TW* (B) and heat killed *TW* (C) followed by staining with rabbit anti-*TW* followed by donkey-anti-rabbit-Alexa647.

Suppl. figure S9: Impact of heat inactivation of *T. whipplei* on their capability to impact barrier function. HT-29/B6 cells were exposed to either *TW* or heat-inactivated *TW* (100°C, 90 min). Only viable *TW* reduced the transepithelial resistance. Median and first and third quartiles, n=5-6. Significance levels were corrected for multiple comparisons. **$p<0.01$, ***$p<0.001$, ns: not significant.

Suppl. figure S10: *T. whipplei* uptake is more frequently found in PARP-positive apoptotic Caco-2 cells. Caco-2 cells were incubated with vital *TW* followed by staining with rabbit anti-*TW* followed by donkey-anti-rabbit-Alexa647 and the apoptosis marker anti-PARP-PE. **A:** Example of a flow cytometric analysis of uptake of *TW* by PARP positive (Q2) and PARP

negative (Q3) cells after gating on Caco-2 and *TW*-positive cells. **B:** Summary of 3 independent experiments of the uptake of *TW* by PARP positive and PARP negative Caco-2 cells. Single values, median and minimum to maximum. ***p<0.001.

Suppl. figure S11: Scheme of co-culture model comprising filter-seeded Caco-2 cells, macrophages, and apically added *T. whipplei*. Hanging filter inserts were placed in 12-well plates with the lower filter side facing up. Caco-2^{Actin} cells (and, for controls, non-transfected Caco-2 cells) were seeded on the lower filter side and were allowed to attach for 8 h. Filter inserts were then transferred to a 24-well plate (now with the upper filter side facing up). On culture day 14, macrophages were seeded to the lower compartment and *TW* was added to the apical compartment (= infection via the basolateral side of the Caco-2 cells).

Suppl. figure S12: Passage of *T. whipplei* into the macrophage compartment through the epithelial layer, as revealed by the co-culture model of Caco-2 cells and primary human macrophages **(Fig. S11)**. **A:** When Caco2^{Actin} cells were used as the filter-based intestinal epithelial cell (IEC) layer, macrophages (MF) attached to the well bottom were HLA-DR-positive and contained epithelial actin-GFP as well as *TW*-positive material. **B:** IEC layer with Caco-2 cells not expressing actin-GFP. Macrophages only display *TW*-positive material. **C:** Exposure of Caco2^{Actin} cells with *TW* in the absence of macrophages. IECs express actin-GFP, but not HLA-DR. Images depicted are representative of two experiments. Size bars, 20 µm.

Suppl. figure S13: Establishment of the flow cytometric analysis of uptake of *T. whipplei* by Caco-2 cells. Caco-2 cells were incubated with vital *TW* and stained for flow cytometric analysis with **A:** rabbit serum and donkey- anti-rabbit-Alexa647 as isotype control or **B:** rabbit anti-*T. whipplei* followed by donkey-anti-rabbit-Alexa647.

Suppl. video S1: Time series of live cell *TW* exposition (single cells). Colocalization (arrows) of *TW* rods (green) with caveolin (caveolin-mRFP, red).

Suppl. video S2: Z-stack of live cell *TW* exposition (monolayer). Colocalization (arrows) of *TW* rods (green) with caveolin (caveolin-mRFP, red).

Suppl. video S3A: Co-expression of cav1-GFP (green) and D82-cav1-mRFP (red) to show functional defect of D82-cav1. **B:** Detail of suppl. video 3A.
