# Supplementary figures and images for: Uptake of Tropheryma whipplei by Intestinal Epithelia
Source: Int J Mol Sci. 2023 Mar 24;24(7):6197. doi: 10.3390/ijms24076197 (PMC10094206; doi:10.3390/ijms24076197)

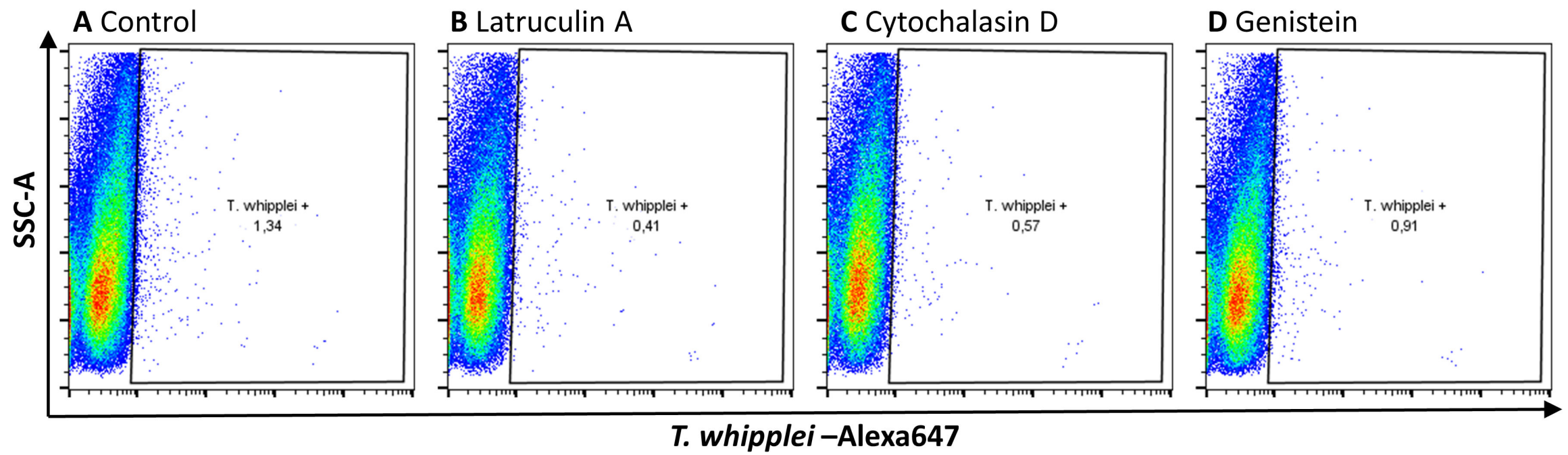

Supplement: Supplementary file 1 [file ijms-24-06197-s001.zip › S1.pdf]

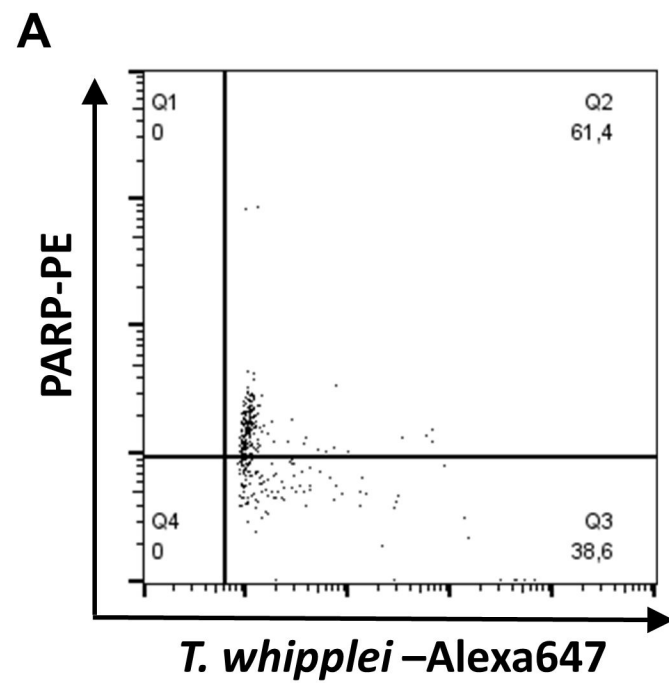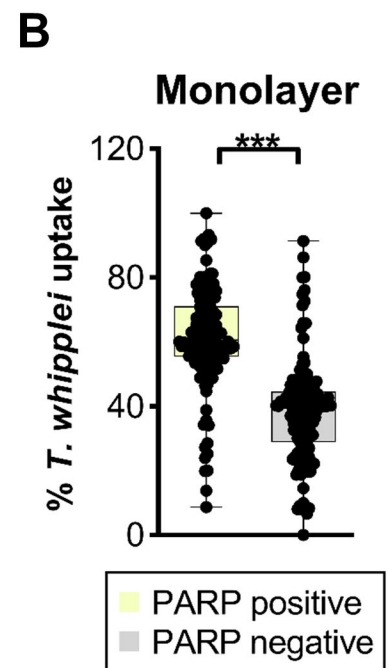

Supplement: Supplementary file 1 [file ijms-24-06197-s001.zip › S10.pdf]

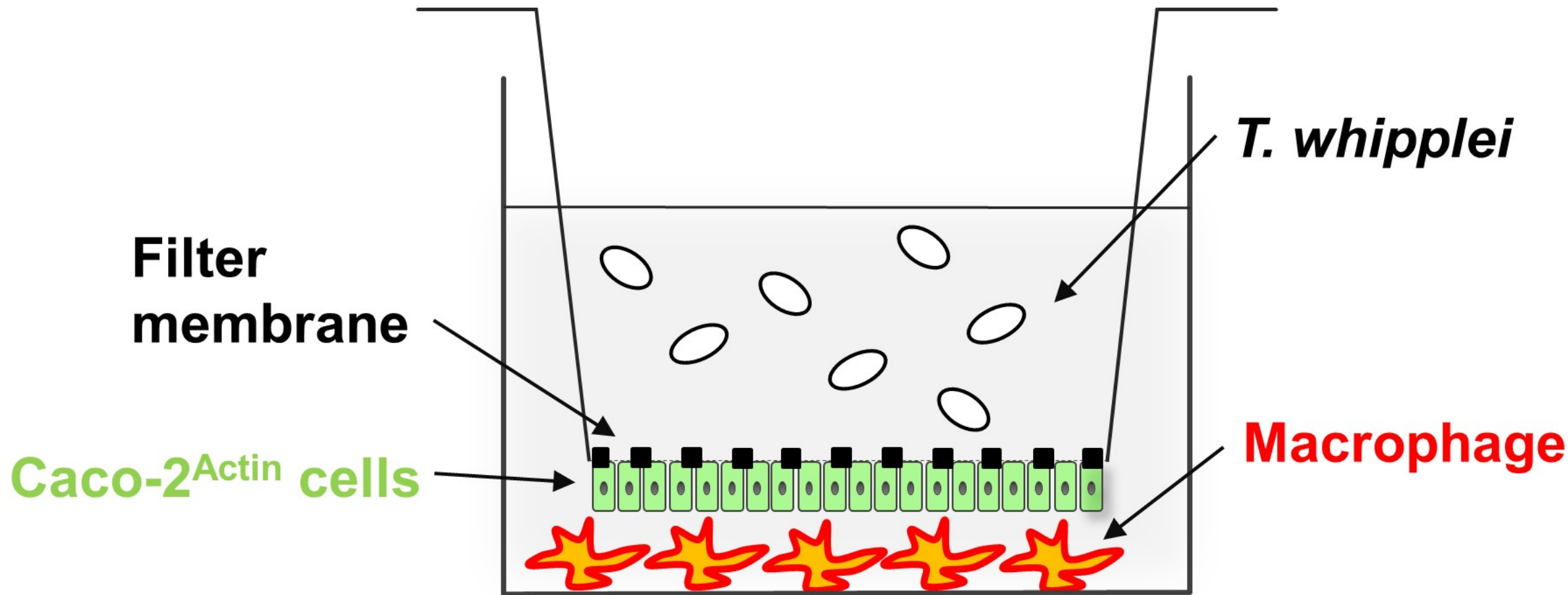

Supplement: Supplementary file 1 [file ijms-24-06197-s001.zip › S11.pdf]

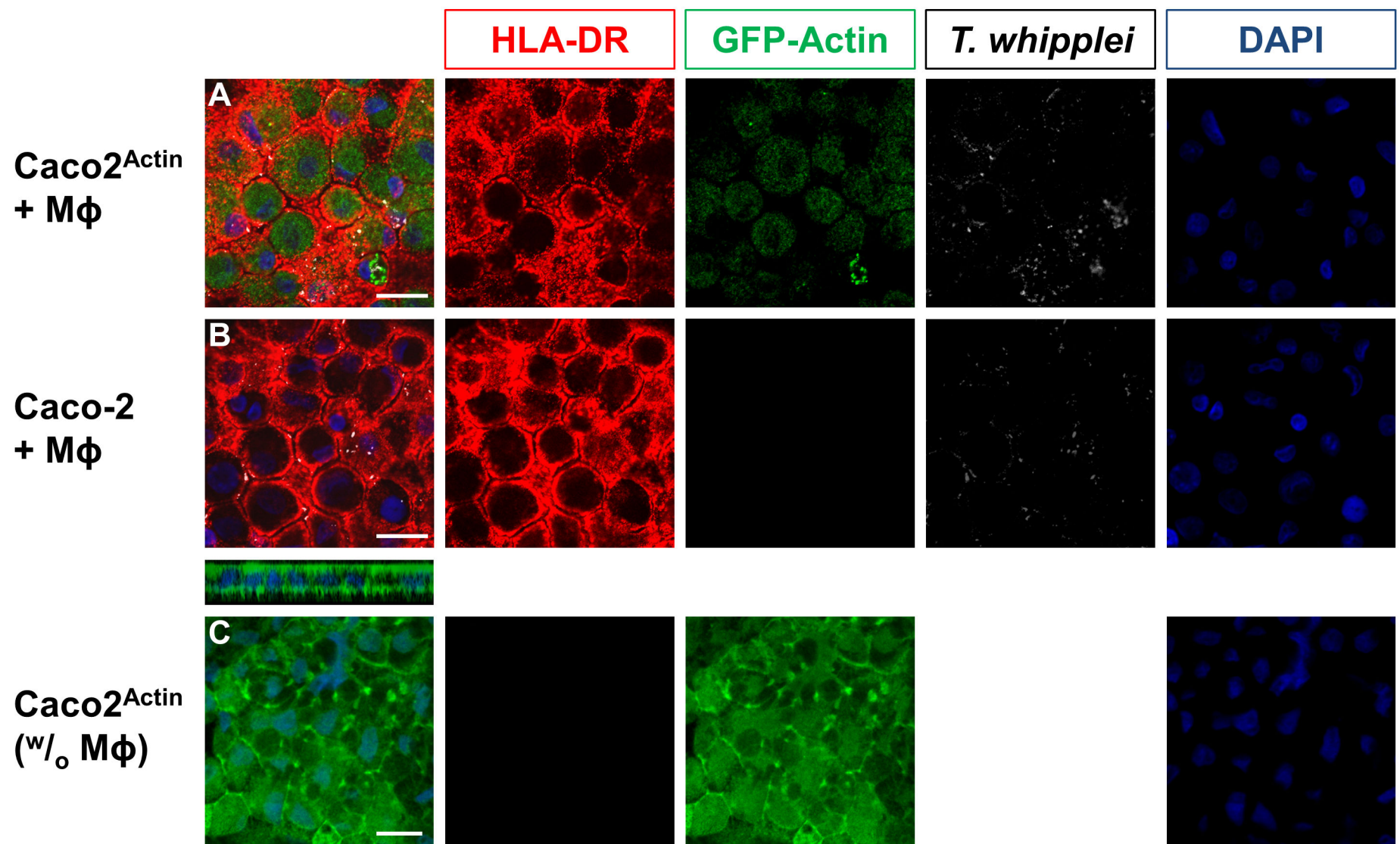

Supplement: Supplementary file 1 [file ijms-24-06197-s001.zip › S12.pdf]

**A** Isotype Control

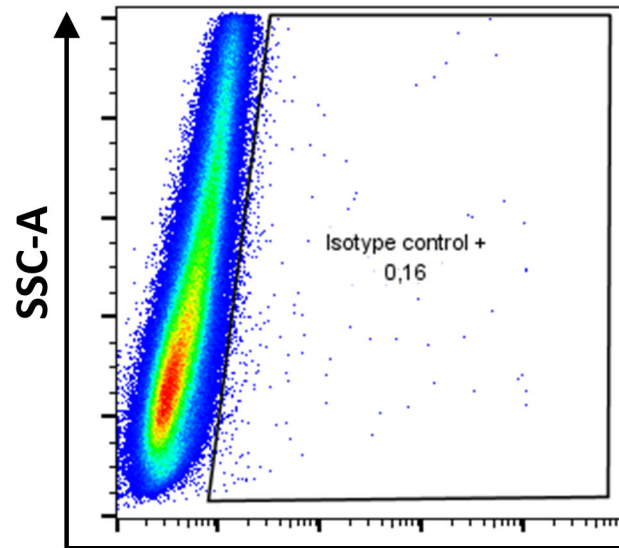

**B** Rabbit anti *T. whipplei*

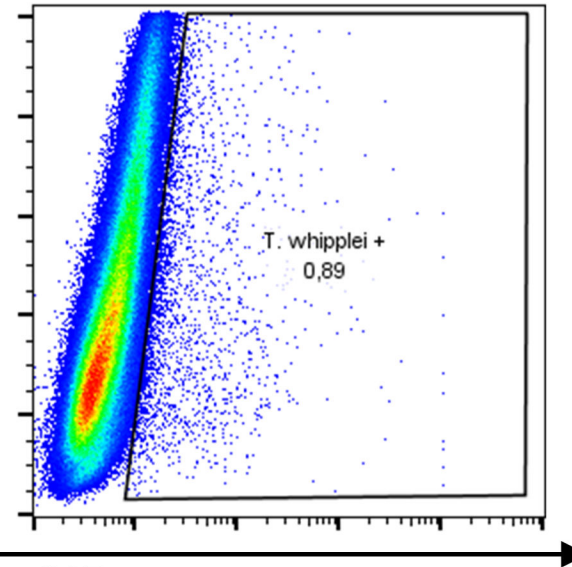

Alexa647

Supplement: Supplementary file 1 [file ijms-24-06197-s001.zip › S13.pdf]

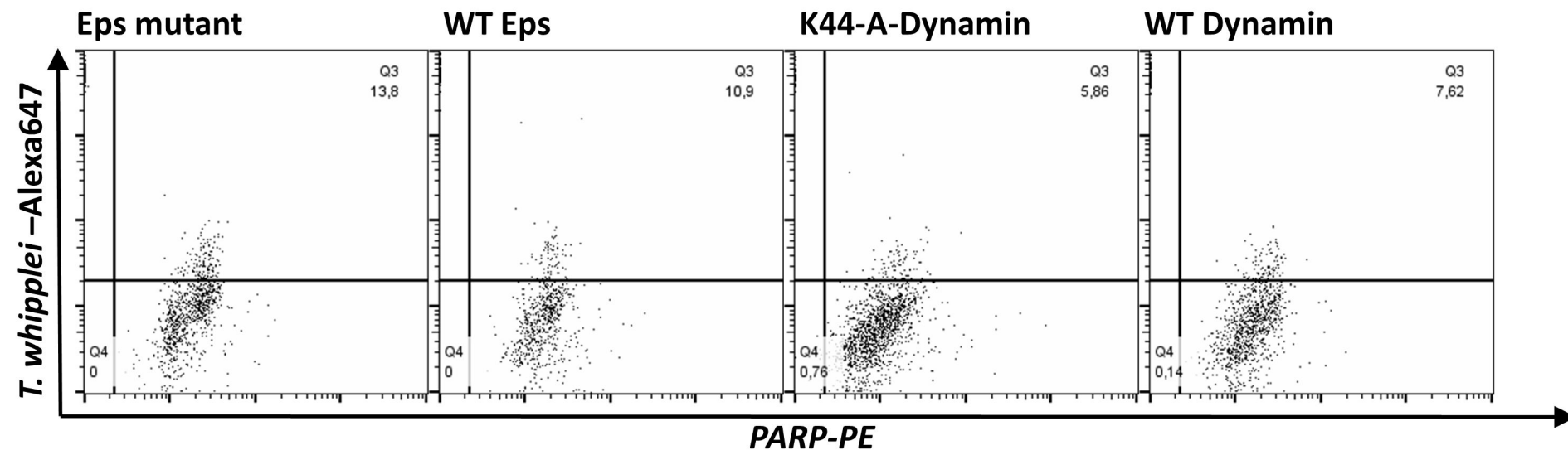

Supplement: Supplementary file 1 [file ijms-24-06197-s001.zip › S2.pdf]

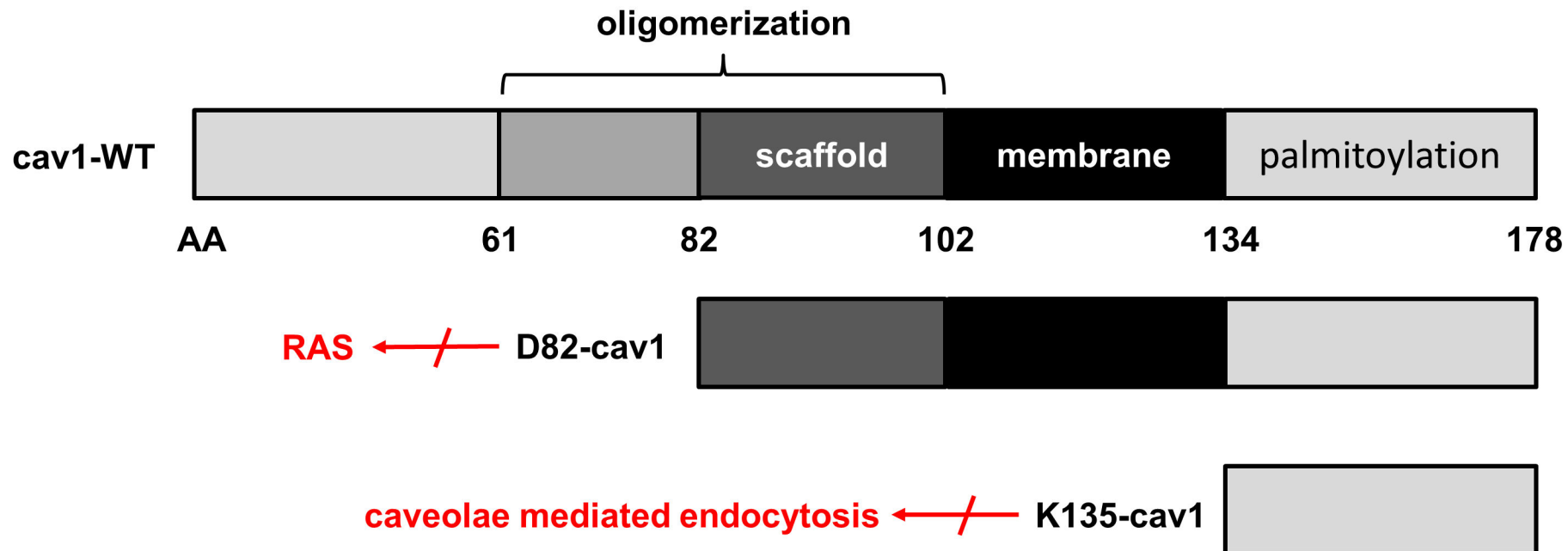

Supplement: Supplementary file 1 [file ijms-24-06197-s001.zip › S4.pdf]

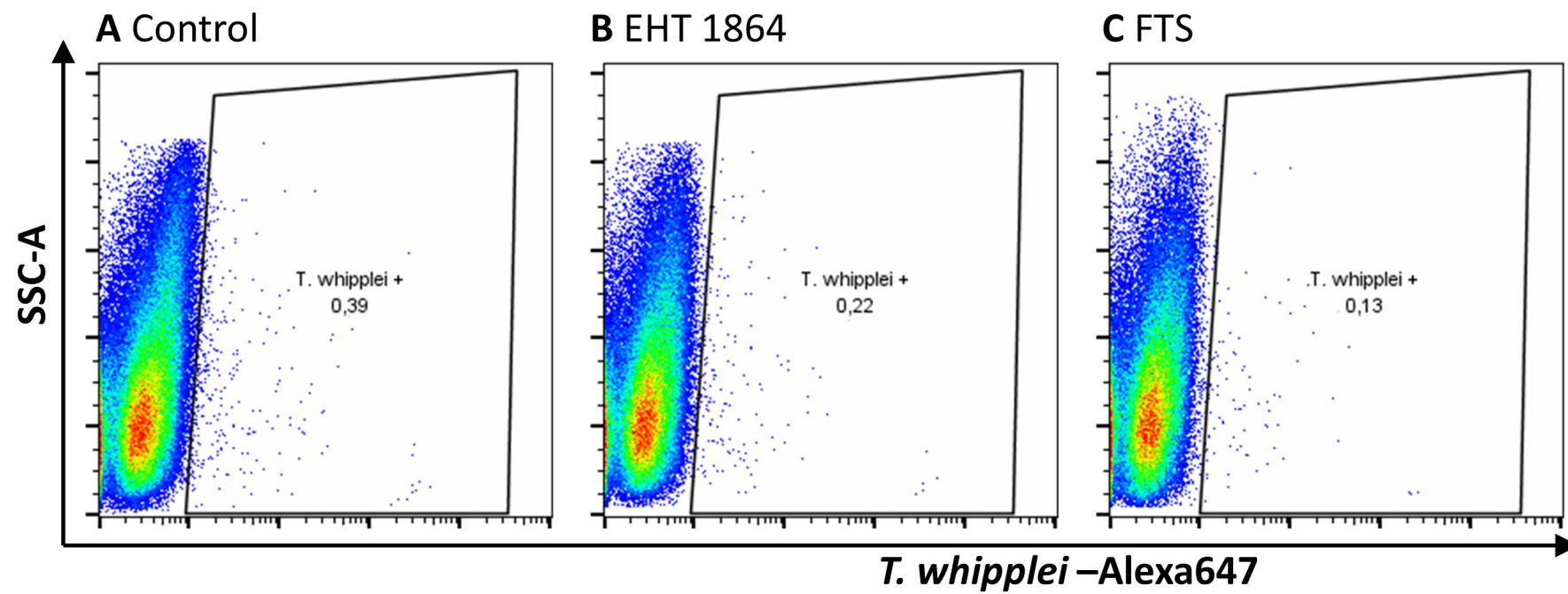

Supplement: Supplementary file 1 [file ijms-24-06197-s001.zip › S5.pdf]

***T. whipplei*:**      no    yes   yes   no   yes   yes   no   yes   yes

caveolin-1

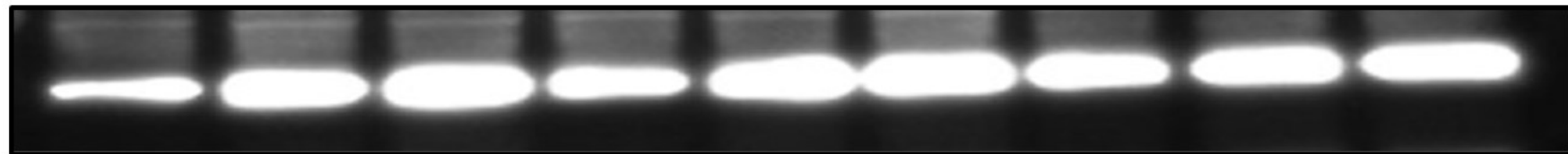

caveolin-2

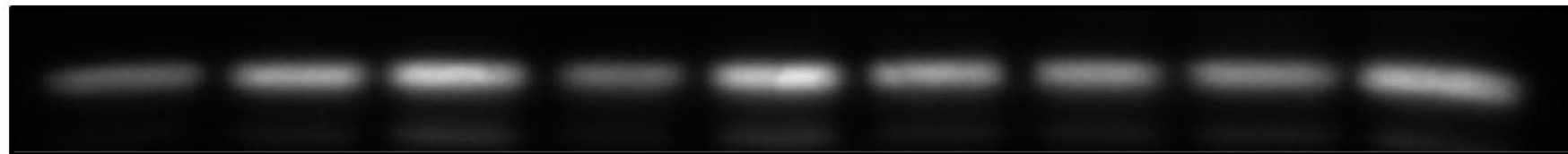

actin

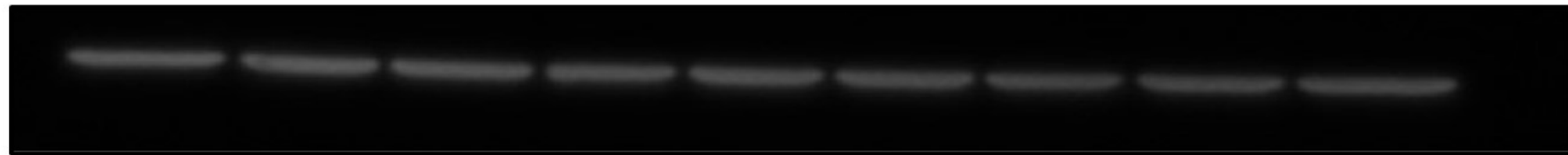

Supplement: Supplementary file 1 [file ijms-24-06197-s001.zip › S6.pdf]

# Monolayer

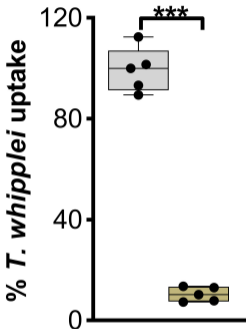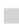

*T. whipplei*

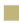

Heat inactivated

Supplement: Supplementary file 1 [file ijms-24-06197-s001.zip › S7 - Copy.pdf]

# Monolayer

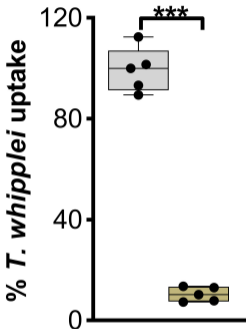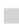

*T. whipplei*

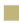

Heat inactivated

Supplement: Supplementary file 1 [file ijms-24-06197-s001.zip › S7.pdf]

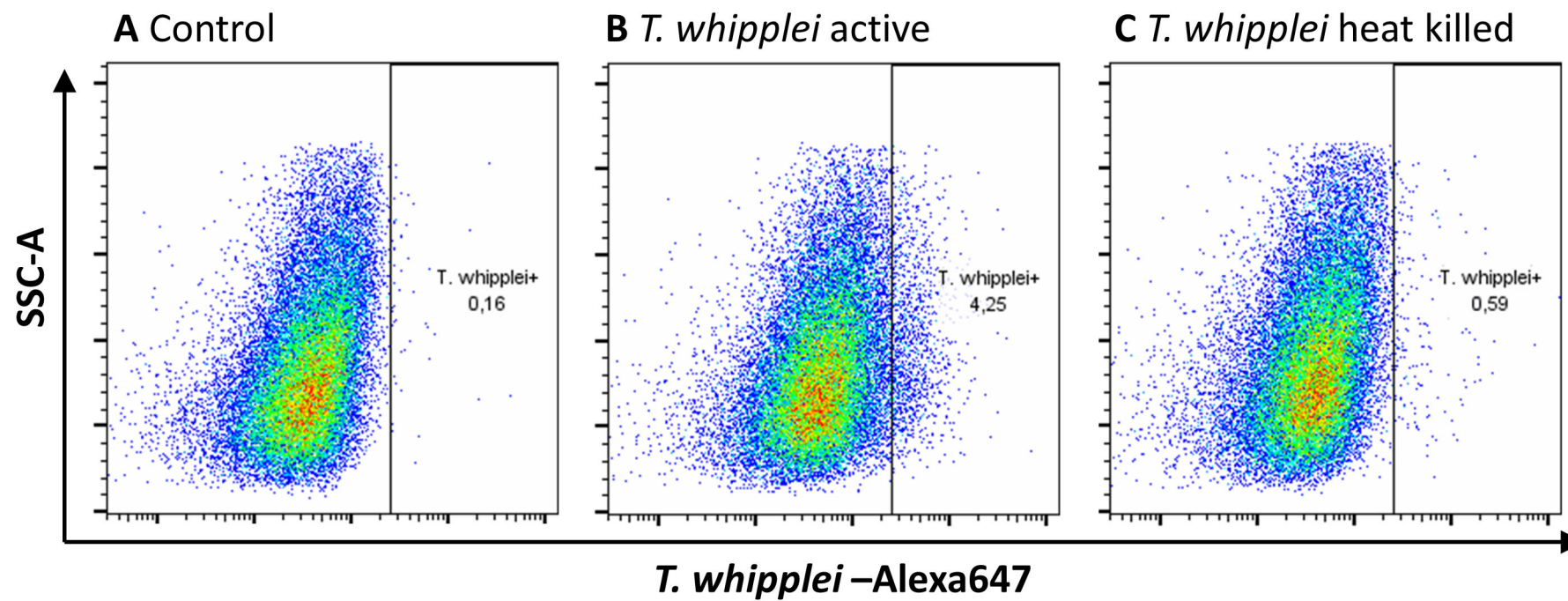

Supplement: Supplementary file 1 [file ijms-24-06197-s001.zip › S8.pdf]

# Monolayer

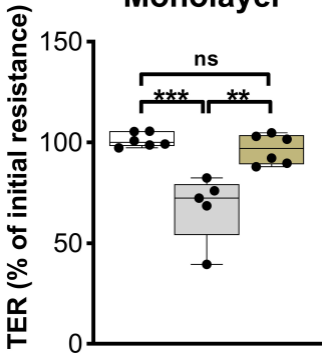

□ control

■ *T. whipplei*

■ Heat inactivated

Supplement: Supplementary file 1 [file ijms-24-06197-s001.zip › S9.pdf]
